# Crop Load Links Gustatory and Feeding Responses to Foraging Specialization in Honey Bees

**DOI:** 10.3390/insects17100980

**Published:** 2026-09-22

**Authors:** Emilia A. Moreno Coellar, Pilar A. Fernández, Micael A. Roldán, Andrés Arenas

**Affiliations:** 1 Grupo de Fisiología del Comportamiento y Sociobiología de Abejas, Departamento de Biodiversidad y Biología Experimental, Facultad de Ciencias Exactas y Naturales, Universidad de Buenos Aires, Buenos Aires C1428EGA, Argentina; pilar.fernandez10@uba.ar (P.A.F.); micael@uba.ar (M.A.R.); 2 Instituto de Biodiversidad y Biología Experimental y Aplicada (IBBEA), CONICET, Universidad de Buenos Aires, Buenos Aires C1428EGA, Argentina

**Keywords:** crop content, division of labour, forager workers, nutritional physiology, proboscis extension reflex (PER), sucrose sensitivity

## Abstract

Honey bee foragers specializing in nectar, pollen, and water collection differ in their physiological demands and in how the collected resource is transported. Here, we investigated whether physiological and behavioral differences among forager types are associated with crop load, the amount of solution carried in the crop. Alternatively, we tested whether crop sugar concentration was more closely related to foraging specialization. We compared sucrose responsiveness, sucrose ingestion, crop load, and crop sugar concentration in nectar, pollen, and water foragers at the beginning and end of the foraging visit. At the beginning of the visit, pollen foragers carried the heaviest crop loads with the highest sugar concentrations and showed the lowest sucrose responsiveness and ingestion, whereas nectar foragers carried lighter crop loads with intermediate sugar concentrations and showed higher responsiveness and ingestion. Water foragers exhibited the lightest and most dilute crop contents and the highest sucrose responsiveness and ingestion. By the end of the visit, nectar foragers showed reduced responsiveness and ingestion as their crop loads increased, whereas pollen foragers showed the opposite pattern as their crop loads decreased. Notably, water foragers also showed reduced sucrose responsiveness and ingestion as their crop loads increased, despite a marked decrease in crop sugar concentration. In water foragers, crop load and crop sugar concentration changed in opposite directions, with behavioral responses consistently decreasing as crop load increased. Overall, these findings support a link between crop load and foraging specialization and suggest a role for crop distension in modulating sucrose responsiveness and feeding motivation in honey bees.

## Simple Summary

Honey bees collect different resources according to the needs of their colony, and different groups of foragers specialize in collecting nectar, pollen, or water. We show that these specializations are associated with the physiological state of the bees and with how much food is carried in their crop. As the crop fills during nectar and water collection, but depleted during pollen foraging, crop load provides information that helps regulate sugar-related responses such as sucrose sensitivity or feeding. These findings reveal that internal physiological signals may coordinate the collection of different resources and contribute to the regulation of resource acquisition at the colony level.

## 1. Introduction

Division of labour is one of the defining attributes of social insects and underlies their ecological success. In honey bee colonies, workers perform different tasks throughout their lives, becoming nectar, pollen, or water foragers at around the second week of adult life [1,2,3]. Division of labour can be explained by variation in response thresholds to task-related stimuli [2,4,5], whereby individuals with the lowest thresholds are more likely to be recruited to perform a given task. Task specialization, however, is graded rather than fixed: individuals can shift between tasks over their lifetime, and some foragers collect more than one resource within a single trip (e.g., nectar and pollen). Because sucrose is the primary component of nectar, variation in sucrose responsiveness has been extensively studied in relation to foraging specialization.

Differences in sucrose responsiveness among nectar and pollen foragers are influenced by genetic factors, as demonstrated by the selection of honey bee strains exhibiting high and low pollen-hoarding behavior [6]. However, sucrose responsiveness is also influenced by transient changes in the internal physiological state; e.g., it increases after feeding or stress [7]. Sucrose responsiveness also varies throughout the foraging visit, with pollen and nectar foragers showing opposite patterns when tested upon arrival and departure from the food source [8]. When bees are tested upon return to the hive after a successful foraging trip, pollen foragers generally respond to lower sucrose concentrations than nectar foragers [9,10], yet this pattern reverses upon arriving at the foraging site [8].

Nectar is a complex solution [11] containing water and a mixture of sugars, including sucrose, glucose, and fructose [3,12], which provide energy for all colony members. Nectar is transported internally in the crop [13], a specialized foregut compartment that expands as nectar is collected and stored. Here, we use crop load to refer to the amount of solution contained in the crop and crop sugar concentration to the concentration of sugars in these contents. The crop is connected to the proventriculus, a valve that regulates the passage of food into the midgut for digestion, thereby helping to maintain stable haemolymph sugar levels when circulating carbohydrate levels decrease [14,15]. In contrast, pollen primarily supplies proteins, lipids, vitamins, and minerals [16] required for brood rearing and the nutrition of young bees and the queen. Although the sugar content of pollen can be substantial, foragers do not consume pollen at the collection site. Pollen is collected in the corbiculae (or pollen baskets) located on the outer surface of the hind tibiae. Foragers use a nectar load carried in the crop to moisten and pack pollen grains into the corbiculae. For these reasons, pollen foraging is expected to result in a negative carbohydrate balance over the course of a foraging trip, accompanied by a progressive decrease in crop load.

Differences in crop load among pollen and nectar foragers have been consistently reported. Pollen foragers leave the hive carrying heavier crop loads than nectar foragers [17,18,19,20], a pattern also observed in stingless bees [21]. These differences may be related to the energetic demands associated with collecting each type of resource and to the way in which these resources are transported. While nectar foragers face a trade-off between carrying crop contents and preserving crop capacity for nectar collection, pollen foragers are not subject to the same constraint because pollen is transported externally [17,18,19,20].

In addition to nectar and pollen, water is essential for brood food production, nest cooling, and colony homeostasis [3,22,23,24,25,26]. Like nectar, water is transported internally in the crop; however, it provides no metabolic fuel to the collecting worker and instead dilutes the sugar content of the crop [27]. Water foragers therefore offer a unique opportunity to investigate how crop load status is associated with task specialization, because water collection increases crop load while decreasing its sugar content.

Previous evidence linking crop load to gustatory responsiveness comes from an early characterization of water foragers [7]. Sucrose responsiveness declined after water collection, while the volume expelled from the crop increased markedly, suggesting an association between crop filling and gustatory sensitivity. This raises the broader question of whether water, nectar, and pollen foragers can be positioned along a common gradient of crop load and whether their sucrose responsiveness and ingestion vary accordingly. An open question is whether these behavioral responses are more closely associated with crop load or with crop sugar concentration.

The aim of this study was to investigate whether foraging role is associated with crop load, regardless of crop sugar concentration and to what extent it relates to gustatory and feeding responses in honey bee foragers. Building on previous knowledge of the physiological profiles of nectar and pollen foragers [8,28], we extended our study to water foragers, whose collection of a non-energetic resource transported internally in the crop provides a useful comparison for examining whether crop load or crop sugar concentration relate to foraging specialization.

### Study Hypotheses and Predictions

We hypothesized that foraging specialization is associated with crop load. Accordingly, we expected pollen, nectar, and water foragers to differ in the amount of liquid food carried in the crop, with corresponding differences in sucrose responsiveness and ingestion across the foraging visit.

Consistent with the crop load hypothesis, at the beginning of the visit, we predicted that pollen foragers would carry heavier crop loads and show lower sucrose responsiveness and ingestion than nectar foragers. Conversely, nectar foragers were expected to carry lighter crop loads and show higher sucrose responsiveness and ingestion than pollen foragers. Water foragers were predicted to show an even more pronounced profile than nectar foragers, with lighter crop loads and higher sucrose responsiveness and ingestion.

At the end of the visit, we predicted that each forager type would show changes in crop load relative to the beginning of the visit, accompanied by corresponding changes in sucrose responsiveness and ingestion. Nectar foragers were expected to return with heavier crop loads as a result of nectar collection, together with lower sucrose responsiveness and ingestion. In contrast, pollen foragers were expected to return with lighter crop loads, consistent with the use of this initial provision as an energy source during foraging, together with higher sucrose responsiveness and ingestion. Water foragers were expected to return with heavier crop loads together with lower sucrose responsiveness and ingestion.

Alternatively, the crop sugar concentration hypothesis suggests that foraging specialization is related to sugar availability as reflected by its concentration in the crop. While in most cases we expected the behavioural profiles of the different forager types to follow the pattern described above, it predicts the highest concentrations in pollen foragers, intermediate concentrations in nectar foragers, and the lowest concentrations in water foragers at the beginning of the foraging visit, with the opposite pattern for sugar concentration at the end of the visit for pollen and nectar foragers. For water foragers, however, the hypothesis predicts the lowest crop sugar concentration at the end of the visit, which should be associated with increased sucrose responsiveness and ingestion, rather than the decreased responses predicted by the crop load hypothesis. Thus, water foragers provided a particularly informative case in which crop load and crop sugar concentration were expected to change in opposite directions across the foraging visit, potentially resulting in contrasting behavioural outcomes.

## 2. Methods

We tested European honey bees *Apis mellifera ligustica* Spinola, 1806, during the February–March 2025 foraging season. Foragers were trained to visit a foraging station located 40 m from the apiary, near a natural water source. The foraging station consisted of ad libitum feeders containing 15% sucrose–water solution and dry, bee-collected multifloral pollen that was ground before being offered. The feeders were placed separately (20 cm apart) on a wooden platform (30 × 40 cm). The natural water source was replenished daily using the city water supply to prevent it from drying out. Foragers were sampled according to forager type (pollen, nectar, or water) and phase of the foraging visit (beginning or end), resulting in six experimental groups: (i) pollen-beginning (PB), (ii) pollen-end (PE), (iii) nectar-beginning (NB), (iv) nectar-end (NE), (v) water-beginning (WB), and (vi) water-end (WE). Bees sampled at the beginning and end were independent individuals; each bee was collected only once and was not released after the experiment.

Our study was designed as a comparative experiment, with the relevant contrasts being among the three forager types (pollen, nectar, and water) and between the beginning and end of the foraging visit. Within each experiment, all groups were tested using the same standardized procedure, allowing direct comparisons across forager types and foraging phases.

### 2.1. Experiment 1: Sucrose Responsiveness

In this experiment, we quantified sucrose responsiveness of pollen, nectar, and water foragers at the beginning and end of the foraging visit, as a measurement of sugar sensitivity associated with crop load or crop sugar concentration (see Section Study Hypotheses and Predictions). In the laboratory, we chilled foragers in a refrigerator (−5 °C) and carefully restrained them in metal tubes, allowing their mouthparts to move freely [29,30]. We offered them water until satiation and then placed them in the incubator [33 °C, 60% relative humidity (RH) and darkness] for 20 to 30 min. We evaluated sucrose responsiveness by means of the proboscis extension reflex (PER); an innate response triggered after touching the bee’s antennae with a sufficiently concentrated sucrose solution [31]. We stimulated the antennae of restrained bees with a series of sucrose-water solutions of increasing concentrations (0.1, 0.3, 1, 3, 10 and 30%) to determine which solution elicited the extension of the proboscis. This ensured that the observed responses were genuine and not due to a lack of motivation or sensory impairment. Bees were not fed during the trials to avoid altering their motivation to respond. Bees were tested in sequential order, starting from the lowest to the highest concentration of sucrose solution with an inter-trial interval of 2 min. Water was provided before the first sucrose stimulus and between sucrose trials to standardize hydration status and minimize the potential influence of thirst on sucrose responsiveness [4,5]. Bees that responded to water more than twice, including the initial water presentation and/or subsequent water presentations between sucrose trials, were excluded from the final analysis.

The concentrations of all sucrose solutions in this study were prepared and reported as *w*/*w* sucrose solution: weight (g) sucrose/(weight (g) sucrose + (g) water).

### 2.2. Experiment 2: Sucrose Ingestion

In this experiment, we quantified sucrose ingestion of pollen, nectar, and water foragers at the beginning and end of the foraging visit, as a behavioural measure of feeding motivation associated with crop load and crop sugar concentration (see Section Study Hypotheses and Predictions). Each bee was isolated and placed inside a modified 10-mL plastic syringe fitted with a mesh at one end with a plastic tip. The syringes were kept inside a box for 10 min before measurements. Each bee was then allowed to feed for 20 min from the tip containing a sucrose solution of known concentration (0%, 3%, or 30%). We removed the tip from the syringe and fitted it onto a micropipette to displace the remaining liquid. Sucrose ingestion was calculated as the difference between the initial volume (80 µL) and the volume recovered from the tip by adjusting the plunger of the micropipette [32]. The resulting values were converted to milligrams (mg) by incorporating the density of each sucrose solution (0%, 3%, and 30% *w*/*w*; 0.998234, 1.009934, and 1.126984 g mL^−1^, respectively, standard reference densities tabulated at 20 °C [33]), allowing for accurate conversion from volume to mass.

### 2.3. Experiment 3: Crop Load and Sugar Concentration

In this experiment, we quantified crop load (mg) and crop sugar concentration (% *w*/*w*) in pollen, nectar, and water foragers at the beginning and end of the foraging visit, as potential proximal mechanisms that relates to foraging specialization (see Section Study Hypotheses and Predictions). Foragers at the beginning of the visit were collected at the foraging station before resource collection, whereas foragers at the end of the visit were collected upon return to the hive. In the laboratory, we dissected each individual to extract the crop, following a modified version of the protocol described by Carreck et al. [34]. While the original method relies on dissecting the unmounted bee via bilateral incisions to extract the entire gastrointestinal tract and associated glands, our approach was specifically optimized for targeted crop isolation. Briefly, a freshly dead forager was prepared by removing its wings and legs. To ensure firm anchorage during the procedure, we secured the specimen in a small pool of melted wax using a cauterizer and placed it under a stereomicroscope (Leica EZ4, Wetzlar, Germany). Instead of cutting both lateral sides of the abdomen, we made a single incision on the right side. One blade of fine scissors was inserted under the overlapping edge of the fifth abdominal tergite (A5), keeping the inner blade parallel to the body wall to avoid internal damage. The incision was continued across the tergite. The dorsal abdominal wall was then gently lifted with a fine needle until completely detached, allowing us to carefully extract only the crop. The isolated organ was placed on a pre-weighed microscope slide and weighed using a precision analytical balance (PR Series, OHAUS Corporation, Parsippany, NJ, USA; readability: 0.0001 g). Whenever possible, the sugar concentration of the crop contents was measured using a handheld analog refractometer (A. KRÜSS Optronic GmbH, Hamburg, Germany) and expressed as percent weight per weight (% *w*/*w*) [35,36,37].

### 2.4. Statistical Analysis

All statistical analyses were performed using R version 4.3.3 (R Core Team, Vienna, Austria; https://www.R-project.org/, accessed on 16 September 2026).

#### 2.4.1. Experiment 1: Sucrose Responsiveness

In experiment 1, when the PER occurred, we assigned values of 1 and when it did not, we assigned values of 0. We analyzed the proportion of PER by means of a multiplicative generalized linear mixed model assuming a Bernoulli distribution. We used the ‘glmmTMB’ function of the ‘glmmTMB’ package [38].

We performed a 2-way analysis of variance (ANOVA) where we considered forager type (a three-level factor corresponding to water, pollen and nectar foragers) and foraging phase (a two-level factor corresponding to beginning and end of the foraging visit) as fixed effects. The experimental days were considered as a random effect. We evaluated the model fit with the simulation-based diagnostics from the ‘DHARMa’ package [39], which revealed no concerning pattern in the QQplot for the simulated residuals nor in the residuals vs. fitted plot. Model convergence was assessed using the ‘diagnose’ function from the ‘glmmTMB’ package [38]. No issues were detected with the Hessian matrix or parameter gradients, confirming successful convergence.

The significance of the factors was determined by a Wald test by means of the ‘Anova’ function from the ‘car’ package [40]. Post hoc contrasts were conducted on the model to assess effects and significance between fixed factors using the ‘emmeans’ function of the ‘emmeans’ package [41] with a significance level of 0.05. Adjusted predictions were also generated using the ‘emmeans’ function.

#### 2.4.2. Experiment 2: Sucrose Ingestion

In experiment 2, we analyzed the ingested sucrose weight (mg) by means of a multiplicative generalized linear mixed model assuming a normal distribution. The model was fitted using the ‘glmmTMB’ function of the ‘glmmTMB’ package [38].

We considered forager type (a three-level factor corresponding to water, pollen and nectar foragers), foraging phase (a two-level factor corresponding to beginning and end of the foraging visit), and sucrose concentration (a three-level-factor) as fixed effects. Experimental days were considered as random effects. We included a dispersion formula (‘dispformula’ term in ‘glmmTMB’) to model the residual variance as a function of forager type x foraging phase. We evaluated the model fit with the simulation-based diagnostics from the ‘DHARMa’ package [39], which revealed no systematic pattern in the residuals nor evidence of heteroscedasticity. Model convergence was assessed using the ‘diagnose’ function in glmmTMB. No issues were detected with the Hessian matrix or parameter gradients, confirming successful convergence.

Post hoc contrasts were conducted on the model to assess effects and significance between fixed factors using the ‘emmeans’ function of the ‘emmeans’ package [41] with a significance level of 0.05. Adjusted predictions were also generated using the ‘emmeans’ function.

#### 2.4.3. Experiment 3: Crop Load and Sugar Concentration

In Experiment 3, we analyzed crop load (mg) using a generalized linear mixed model with a lognormal distribution. This approach was chosen due to the strictly positive nature of the data and its right-skewed distribution and because the Gaussian model exhibited deviation from the expected residual distribution in the QQplot of simulated residuals. The model was fitted using the ‘glmmTMB’ function of the ‘glmmTMB’ package [38]. Crop sugar concentration (% *w*/*w*) was analyzed using a generalized linear mixed model with a Gaussian distribution.

We considered forager type (a three-level factor corresponding to water, pollen and nectar foragers), foraging phase (a two-level factor corresponding to beginning and end of the foraging visit) as fixed effects. We included a dispersion formula (‘dispformula’ term in ‘glmmTMB’) to model the residual variance as a function of forager type x moment. We evaluated the model fit with the simulation-based diagnostics from the ‘DHARMa’ package [39] which revealed no concerning pattern in the QQplot for the simulated residuals nor in the residuals vs. fitted plot.

For the concentration analysis, we considered the same fixed effects and dispersion formula. We evaluated the model fit with the simulation-based diagnostics from the ‘DHARMa’ package [39] which revealed no concerning pattern in the QQplot for the simulated residuals nor in the residuals vs. fitted plot.

Model convergence was assessed for both GLMMs using the ‘diagnose’ function in glmmTMB. No issues were detected with the Hessian matrix or parameter gradients, confirming successful convergence. Post hoc contrasts were conducted on both models to assess effects and significance between fixed factors using the ‘emmeans’ function of the ‘emmeans’ package [41], with a significance level of 0.05. Adjusted predictions were also generated using the ‘emmeans’ function.

## 3. Results

Across the three experiments, sucrose responsiveness, sucrose ingestion, and crop contents differed among forager types and across the foraging visit. Overall, nectar and water foragers showed similar temporal patterns, whereas pollen foragers showed an opposite pattern.

For clarity, group abbreviations indicate forager type and foraging phase: PB and PE, pollen foragers at the beginning and end of the visit; NB and NE, nectar foragers at the beginning and end; and WB and WE, water foragers at the beginning and end, respectively. For all figures: different letters indicate statistically significant differences among groups (*p* < 0.05), determined by pairwise comparisons of estimated marginal means (emmeans package; Tukey adjustment) and sample sizes are shown in parentheses or brackets.

### 3.1. Experiment 1: Sucrose Responsiveness

The probability of eliciting the proboscis extension response (PER) increased with the sucrose concentration of the solution offered to all forager groups (Figure 1A). However, response profiles differed among forager types and depended on whether bees were tested at the beginning or at the end of their foraging trip. In line with observed responses, the statistical analysis revealed a significant interaction between forager type and foraging phase on sucrose responsiveness (χ^2^ = 199.706; df = 2; *p* < 0.001).

At the beginning of the foraging visit, 2 of 41 nectar, 4 of 42 water, and 4 of 24 pollen foragers were excluded because they responded to water more than twice during the assay, resulting in final sample sizes of 39, 38, and 20 bees, respectively. At the end of the foraging visit, no bees met this exclusion criterion, resulting in final sample sizes of 36, 31, and 27 bees, respectively. At the beginning of the trip, water foragers exhibited the highest responsiveness, responding to the lowest sucrose concentrations tested (Figure 1A). Although water foragers tended to be more responsive than nectar foragers, this difference was not significant (WB/NB: odds ratio = 1.941, *p* = 0.077). By the end of the trip, sucrose responsiveness had decreased significantly in both groups (WB/WE: odds ratio = 9.1001, *p* < 0.0001; NB/NE: odds ratio = 9.4516, *p* < 0.0001). In contrast, pollen foragers exhibited the opposite pattern, with significant lower responsiveness at the beginning and higher responsiveness at the end of the foraging visit (PB-PE: odds-ratio = 0.0290, *p* < 0.0001).

### 3.2. Experiment 2: Sucrose Ingestion

The volume ingested varied according to forager type, sucrose concentration, and the phase of the foraging trip. Analysis of sucrose ingestion (mg) revealed a significant interaction between forager type, sucrose concentration, and foraging phase (χ^2^ = 54.667; df = 4; *p* < 0.0001, Figure 2). Overall, consumption increased with sucrose concentration, indicating that sugar acts as energetic reward for all forager groups, including water foragers. Nectar and water foragers exhibited similar response patterns, characterized by high consumption at the beginning of the foraging trip and lower consumption at its end. In contrast, pollen foragers displayed the opposite pattern, consuming relatively little at the beginning of the trip and substantially more at the end. Because the response patterns differed across sucrose concentrations, comparisons among forager types and between phases of the foraging trip were performed separately for each concentration.

At 3% and 30% sucrose solutions, water foragers ingested significantly more sucrose solution at the beginning than at the end of the visit (WB–WE 3% = 14.188, *p* < 0.0001; WB–WE 30% = 20.634, *p* < 0.0001 (Figure 2)). A similar pattern was observed in nectar foragers (NB–NE 3% = 20.577, *p* < 0.0001; NB–NE 30% = 27.405, *p* < 0.0001), indicating higher ingestion upon arrival at the resource.

Pollen foragers showed a different pattern. At 3% sucrose, they ingested significantly less solution at the beginning than at the end of the visit (PB–PE 3% = −26.451, *p* < 0.0001), whereas at 30% sucrose no significant differences were detected between phases (PB–PE 30% = 8.152, *p* = n.s.). At this higher concentration, pollen foragers also exhibited the highest overall sucrose intake among all groups.

Water ingestion (0%) showed additional differences among forager types (Figure 2). While nectar and pollen foragers did not differ between the beginning and the end of the visit (NB–NE 0% = −0.923, *p* = n.s.; PB–PE 0% = 0.833, *p* = n.s.), water foragers ingested significantly more water at the beginning than at the end of the visit (WB–WE 0% = 11.278, *p* = 0.0043). In addition, pollen foragers ingested more water than both nectar and water foragers at both phases (NB–PB 0% = −31.348, *p* < 0.0001; WB–PB 0% = −21.973, *p* < 0.0001).

### 3.3. Experiment 3: Crop Load and Sugar Concentration

Crop contents varied according to both forager type and foraging phase. Crop load weight was affected by a significant interaction between forager type and foraging phase (χ^2^ = 347.837; df = 2; *p* < 0.001). At the beginning of the foraging visit, nectar and water foragers carried similar crop loads (NB/WB: ratio = 1.253, *p* = 0.4283), whereas pollen foragers departed the hive with significantly heavier crops than both nectar and water foragers (NB/PB: ratio = 0.3755, *p* < 0.001; PB/WB: ratio = 3.33, *p* < 0.001). Following resource collection, nectar foragers returned with significantly heavier crop loads than water foragers (NE/WE: ratio = 1.7365, *p* < 0.001; Figure 3A).

Crop sugar concentration differed in its pattern from crop load (Figure 3B). At the beginning of the foraging visit, pollen foragers exhibited significantly higher crop sugar concentrations than both nectar and water foragers (NB–PB = −24.74, *p* < 0.0001; PB–WB = 34.71, *p* < 0.0001), consistent with their departure from the hive carrying concentrated carbohydrate reserves. In contrast, water foragers exhibited lower crop sugar concentrations at the end than at the beginning of the foraging visit (WE–WB = −13.70), reflecting the progressive dilution of crop carbohydrates during water collection. Mean crop sugar concentration decreased from 16.38% *w*/*w* in water foragers at the beginning of the visit to 2.68% *w*/*w* at the end, representing an approximately 6.1-fold dilution of the crop content.

## 4. Discussion

This study asked whether crop load is associated with gustatory responsiveness and feeding motivation, which in turn are associated with task specialization in honey bees. By simultaneously comparing pollen, nectar, and water foragers across complementary measures at the beginning and end of the foraging visit, our results revealed a consistent association between crop load, the amount of solution in the crop, and forager type. At the beginning of the visit, pollen foragers carried the heaviest and most sugar-concentrated crop contents and showed the lowest sucrose responsiveness and ingestion, whereas water foragers showed the lowest crop load and the highest responsiveness and ingestion, with nectar foragers occupying an intermediate position. At the end of the foraging visit, pollen and nectar foragers showed opposite changes in crop load, accompanied by corresponding changes in their sucrose responsiveness and ingestion. Water foragers provided a particularly informative case. Consistent with the prediction of the crop load hypothesis they returned with heavier but more dilute crop contents and showed reduced sucrose responsiveness and ingestion. Overall, our results support an association between crop load and foraging specialization and suggest a potential role of crop filling dynamic in modulating sucrose responsiveness and feeding motivation in honey bees.

Sucrose responsiveness is known to be modulated by feeding condition [7], and changes across the foraging trip [7,8,28] may therefore reflect underlying crop-load dynamics. The crop, often referred to as the “social stomach”, serves primarily for nectar transport, but foragers can also consume part of its contents to meet their own metabolic demands and help maintain stable haemolymph sugar levels [14,15]; in most cases, however, bees are thought to complete their foraging visits relying largely on circulating haemolymph sugars alone [14,15]. Because a bee’s carbohydrate state is expected to remain relatively stable while the crop still contains nectar, signals associated with crop distension, potentially mediated by stretch receptor neurons innervating the crop, may contribute to the regulation of sucrose responsiveness and feeding motivation, independently of the metabolic fuel the crop provides [7,10,14,15,42].

Previous studies have shown that pollen foragers are generally more responsive to sucrose than nectar foragers when tested upon return to the hive [4,9,10]. Such differences have been interpreted as adaptive, as reduced sucrose responsiveness among nectar foragers may bias searching for productive sources that provide the colony with a higher energy gain [4,7]. Nevertheless, differences in sucrose responsiveness alone do not fully explain why foragers that are highly responsive to sucrose are prone to collect pollen. More recent studies extend this view by showing that sucrose responsiveness depends critically on the phase of the foraging visit [8], suggesting that sucrose responsiveness reflects a dynamic physiological state rather than a fixed characteristic of each forager type. An earlier comparison of water and pollen foragers [7] already suggested a role for crop load independent of resource energetic value: sucrose responsiveness declined after water collection, despite resource providing no direct caloric gain.

Our results confirm and extend this pattern by simultaneously comparing all three forager types across both phases of the foraging visit, and by adding sucrose ingestion and crop sugar concentration as independent measures. Whereas nectar and water foragers exhibited similarly high responsiveness and ingestion upon arrival at the food source and lower values before returning to the hive, pollen foragers displayed the opposite temporal pattern. These behavioral changes were accompanied by consistent variations in crop contents, suggesting that sucrose responsiveness, ingestion, and crop load may be linked through common physiological processes [17,18,19,20,21].

Exploratory observations on resin foragers provide an additional example of foragers that collect a non-food resource externally (Supplementary Figures S1 and S2). Like pollen, plant resins are transported externally in the corbiculae and therefore do not occupy crop capacity during transport. Although resin is a non-food resource, honey bees use it to produce propolis, which contributes to nest construction, maintenance, and social immunity [43]. Returning resin foragers showed intermediate sucrose responsiveness and consistently low crop loads. Because only returning foragers were examined, this observation remains preliminary and is noted here only as a point of interest for future work directly comparing departing and returning resin foragers.

Water foragers provided an interesting opportunity to examine how this forager type is positioned along the crop load gradient. They occupied the lowest end of this gradient at the beginning of the visit, extending the profile observed in nectar foragers rather than resembling pollen foragers across the traits measured. Water foragers exhibited temporal changes in sucrose responsiveness and ingestion similar to those of nectar foragers, while their crop sugar concentration decreased an approximately 6.1-fold reduction in sugar concentration (i.e., to about one-sixth of the initial value).

This extreme profile at the beginning of the visit raises the possibility that water foragers do not constitute a physiologically distinct category, but rather represent an extension of the nectar-forager profile, in which low crop load status is associated with high sucrose responsiveness and the acceptance of highly diluted resources. The similar temporal changes observed in water and nectar foragers further suggest that their feeding responses may be regulated by common physiological processes. Although subtle, these differences could facilitate the ingestion of water and/or highly dilute solutions. This interpretation does not exclude the possibility that water collection is triggered by other signals evaluated either at the individual level, such as dehydration, or at the colony level, such as thermoregulatory demand. Water foraging is known to increase sharply with nest temperature [22,24], raising the possibility that some water foragers are recruited in response to colony-level cooling demand rather than, or in addition to, individual physiological state.

The low crop loads carried by water foragers at the beginning of the visit may be related to the need to preserve crop capacity for transporting water, in contrast with the external, corbicular transport of pollen. The evolution of corbiculae on the hind legs effectively decoupled pollen transport from crop capacity, potentially allowing pollen foragers to exploit resources over greater distances while departing with relatively heavy crop loads. In contrast, water collection remains constrained by the requirement to carry the resource in the crop, which, together with the low initial crop loads of water foragers documented here, may limit water foraging to sites located relatively close to the nest. Consistent with this constraint, water foragers displaced only 300 m from their foraging station show a sharp decline in homing success relative to nectar foragers under comparable conditions (7% versus 92%, respectively), and this deficit is reversed when displaced water foragers are provided with sucrose solution prior to release, restoring homing success to 94% [27]. This finding suggests that the physiological state associated with the low initial crop loads of water foragers may contribute to constraining their foraging range.

This physiological constraint on water foragers, however, may be offset by a key ecological asymmetry between the two resources. Nectar sources are typically patchy and irregularly distributed in space, sometimes located far from the colony, and often provide low per-flower yields that require visiting many flowers to complete a load. Water sources, by contrast, are frequently available ad libitum at a local scale (e.g., a puddle, dew on leaves, or a permanent water body) and, except in particularly arid environments, tend to occur relatively close to the nest. Under conditions where water availability requires traveling greater distances, water foragers might be expected to depart the nest with larger sucrose reserves in the crop. However, this would reduce the crop capacity available for water collection and result in the delivery of diluted rather than pure water, potentially reducing its efficiency for functions such as nest cooling or honey dilution. Water foraging trips may therefore typically require less extensive movement among resource patches than nectar-foraging trips, as water foragers can fill their crops from a single abundant source. This asymmetry in resource accessibility may allow water foragers to forage effectively despite their low initial crop loads, while also contributing to the relatively restricted foraging range suggested for this group.

Considering the natural asymmetries that may exist among sources of different resources, our experiments were conducted with the foraging stations located at a short distance from the apiary. All three forager types were captured at stations located approximately 40 m from the colony, ensuring that foraging distance was standardized across resource types. Nevertheless, our measurements of crop load status and sucrose responsiveness primarily reflect bees foraging over relatively short distances and may therefore underestimate the energetic demands of nectar and pollen foragers exploiting more distant resources. For nectar and pollen foragers, which may forage at greater distances, longer flights could alter energetic expenditure, thus crop load status, potentially influencing sucrose responsiveness. Thus, our findings should not be directly extrapolated to foragers undertaking substantially longer foraging trips. Similarly, although water foragers are generally expected to operate close to the nest, their physiological state under conditions requiring longer-distance water collection remains unknown. Future studies comparing foragers captured at different distances from the colony, or under experimentally controlled flight distances, would help determine how foraging distance interacts with crop load status and sucrose responsiveness across foraging tasks.

Certainly, crop load status is not the only physiological variable through which bees assess their energetic state. Circulating haemolymph sugar levels [14,15], which we did not measure here, might differ among forager types and independently contribute to sucrose responsiveness and ingestion. However, even if haemolymph sugar titres do not differ among forager types, the differences in crop load observed in this study could be sufficient to explain the behavioural differences among specialized foragers. This would further support the relevance of crop load in explaining these behavioural differences. However, our design cannot rule out an additional contribution from circulating sugars, and direct measurements would help establish their relative roles in sucrose responsiveness, ingestion, and task specialization. Metabolomic approaches quantifying circulating sugars would be particularly well suited to test this possibility, allowing simultaneous comparison of haemolymph sugar profiles across pollen, nectar, and water foragers and complementing the crop-level measurements presented here.

From an adaptive perspective, the physiological profiles observed in each forager type were generally consistent with the nature of the resource they collect. At the beginning of the foraging visit, nectar foragers were responsive to and ingested larger amounts of low- to intermediate-concentration sucrose solutions, which are likely to be commonly encountered in the bees’ foraging environment. In contrast, pollen foragers showed lower sucrose responsiveness and a high feeding response to highly concentrated sucrose solutions. Because such high concentrations are unlikely to be commonly encountered in natural nectar sources, this profile may reduce the likelihood that pollen foragers are attracted to nectar sources in their environment. Similarly, water foragers showed a high propensity to ingest the 0% sucrose solution, consistent with their role as water collectors. However, the occurrence of other responses was less readily explained in terms of foraging motivation. For example, water foragers also readily ingested the 3% and 30% sucrose solutions. These responses may reflect physiological regulation of the bees’ internal state rather than greater motivation to collect nectar. Because our assay, based on ingestion from a syringe, does not fully reproduce the sensory and social context of resource collection, appropriate behavioural responses in this context may also depend on resource-related cues and social feedback that influence foraging decisions at the foraging site and/or inside the hive. An unexpected feature of pollen foragers’ ingestion response was their relatively high intake of the 0% sucrose solution. Given their extremely high crop sugar concentrations, water ingestion under the confined experimental conditions may reflect an attempt to restore the bee’s internal state towards homeostasis.

Our findings provide further insight into the relationship between individual physiology and colony-level organization. Classical models of honey bee foraging emphasized that resource collection is regulated primarily by colony nutritional demands [3], whereas more recent studies have highlighted the importance of the physiological and nutritional condition of individual workers in shaping foraging decisions [44,45]. Rather than representing alternative explanations, our results suggest that these mechanisms may be closely integrated. Colony requirements may be mediated through physiological processes acting at the level of individual workers, with crop load associated with sucrose responsiveness and feeding motivation according to the task being performed. In this context, the particularly low crop load of water foragers at the beginning of the visit is consistent with the hypothesis that regulatory pathways associated with individual physiological state may have been co-opted during the evolution of eusociality to contribute to colony-level resource allocation [46].

Finally, our findings provide a framework for understanding how individual physiological and behavioral responses contribute to colony-level resource provisioning and nutritional regulation. This perspective also has implications for beekeeping practices. For instance, providing food inside the hive may transiently alter the physiological state of foragers and, consequently, their motivation to collect particular resources. Similarly, ensuring access to water sources near hives may be particularly important during periods of drought, when water availability can become limiting. Overall, linking nutritional physiology with foraging specialization may help explain how resource-acquisition efficiency across different tasks is shaped by the energetic state of the forager.

## Figures and Tables

**Figure 1:**
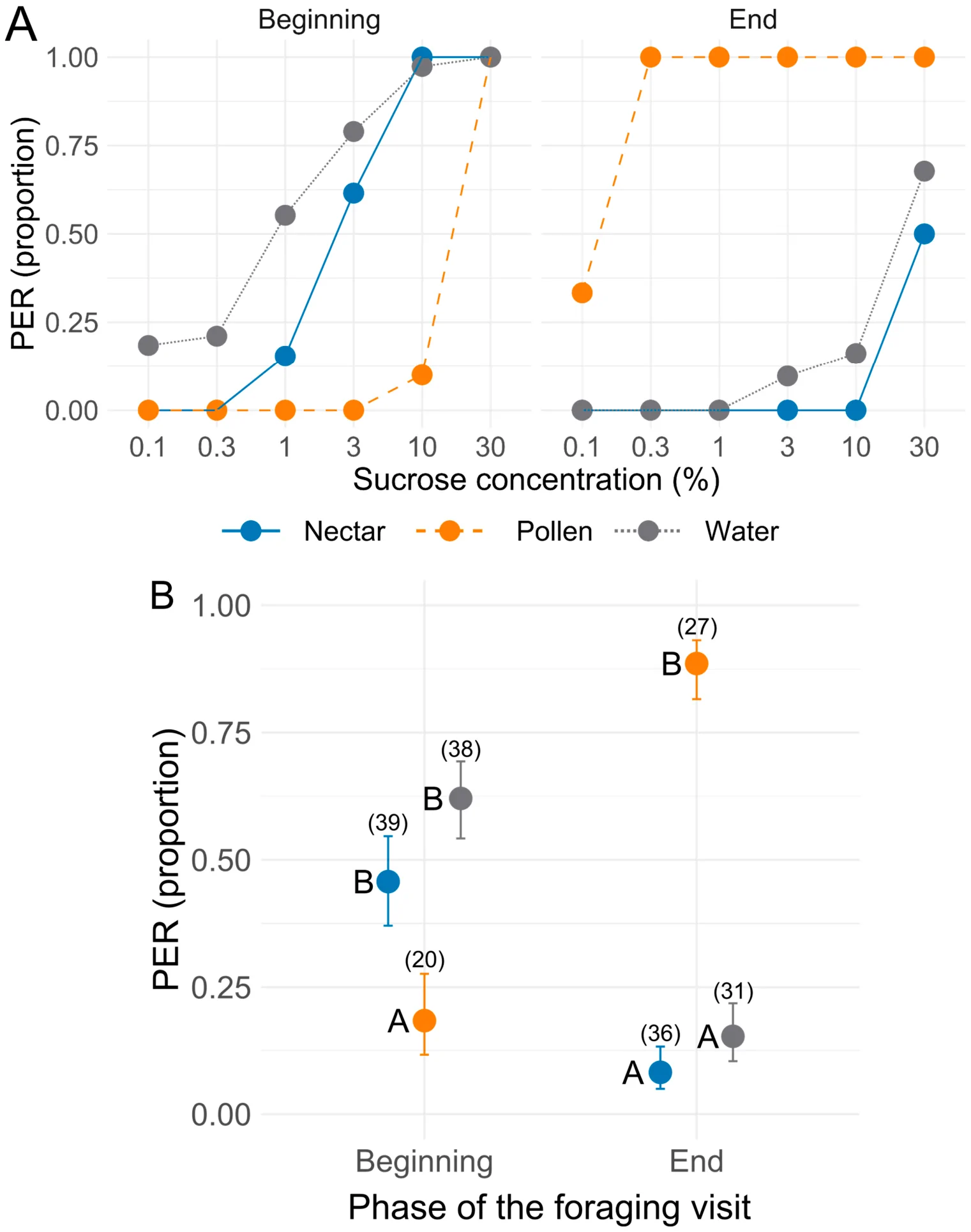
(**A**) Proportion of pollen (orange), nectar (blue), and water (gray) foragers exhibiting the proboscis extension response (PER) to increasing sucrose concentrations when tested at the beginning and end of their foraging trip. (**B**) Model-predicted PER probabilities for pollen (orange), nectar (blue), and water (gray) foragers at the beginning and end of the foraging visit. Circles represent estimated marginal means (EMMs) derived from the generalised mixed model (GLMM), and error bars indicate 95% confidence intervals. Statistical significance was determined using pairwise comparisons of estimated marginal means (emmeans package; Tukey adjustment). Different letters denote statistically significant differences among groups (*p* < 0.05). Sample sizes are shown in parentheses.

**Figure 2:**
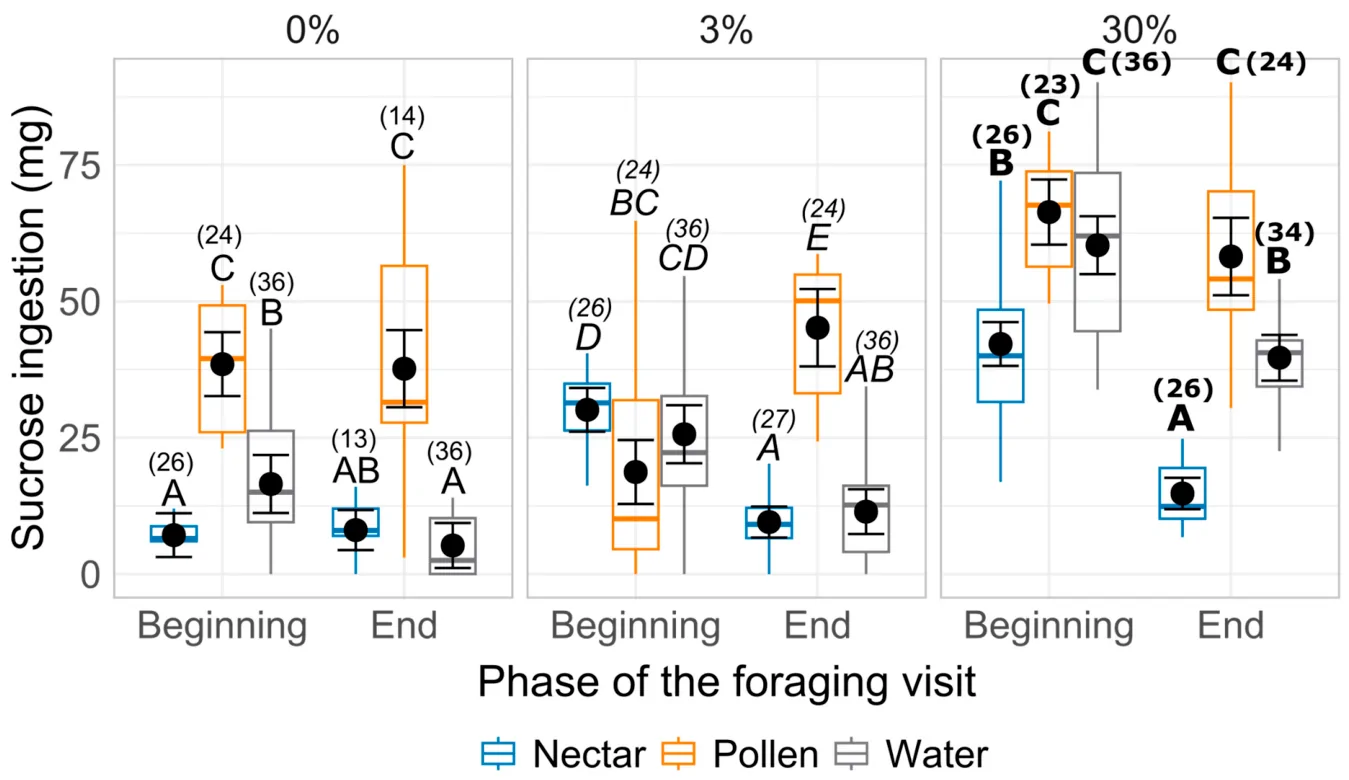
Amount of sucrose solution ingested (mg) by pollen (orange), nectar (blue), and water (gray) foragers in response to increasing sucrose concentrations when tested at the beginning and at the end of their foraging trip. Boxes represent the interquartile range of observed data, with the median indicated by the horizontal line. Black dots denote model-predicted means, and error bars indicate 95% confidence intervals. Statistical significance was determined using pairwise comparisons of estimated marginal means within each sucrose concentration (emmeans package; Tukey adjustment). Different letters indicate statistically significant differences among groups within each sucrose concentration (*p* < 0.05); regular letters indicate comparisons at 0.3%, *italic* letters at 3%, and bold letters at 30%. Sample sizes are shown in brackets.

**Figure 3:**
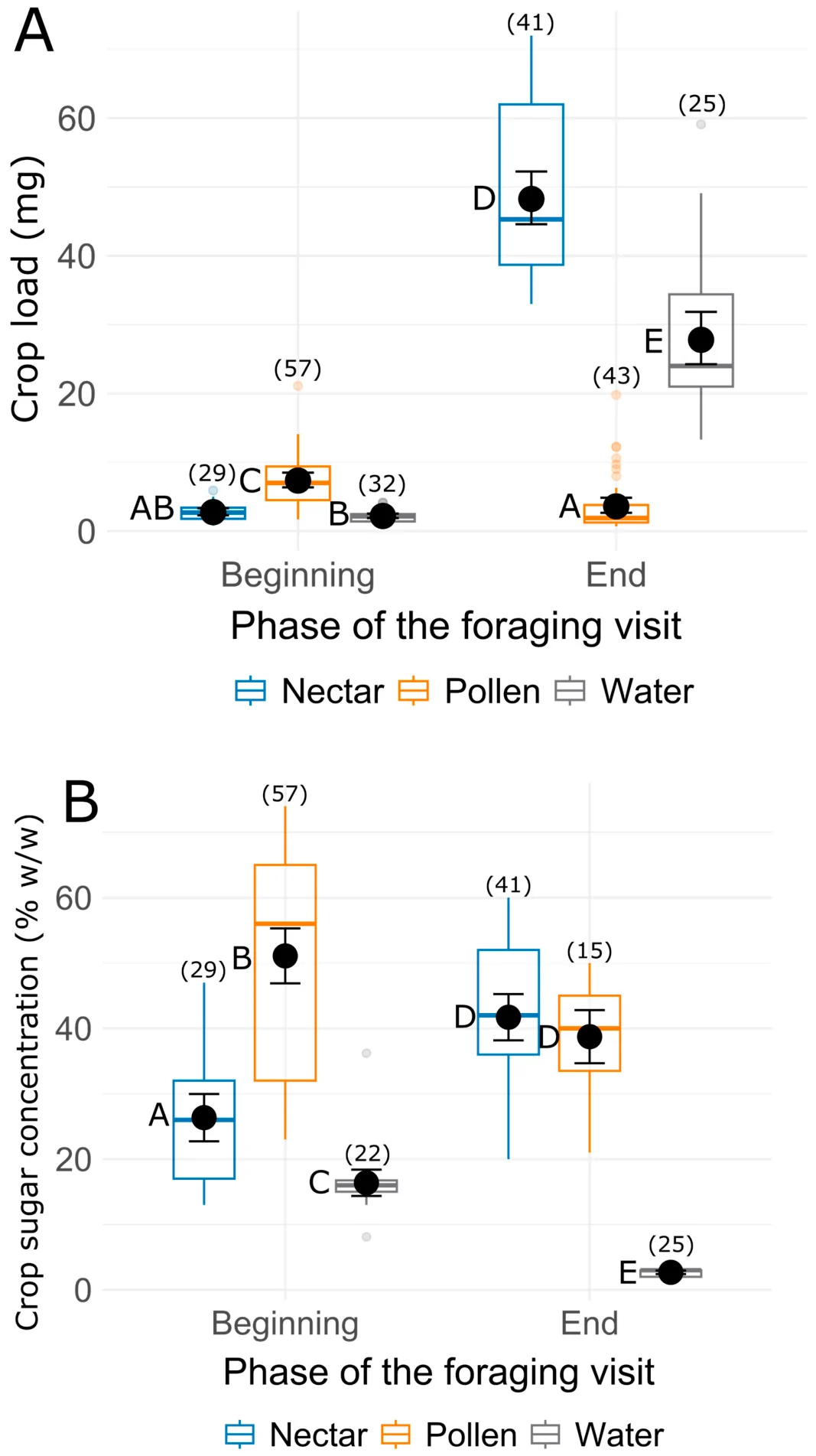
(**A**) Crop load (mg) and (**B**) crop sugar concentration (% *w*/*w*) of pollen (orange), nectar (blue), and water (gray) foragers at the beginning and at the end of their foraging trip. Boxes represent the interquartile range, with the median indicated by the horizontal line. Black dots denote model-predicted means, and error bars indicate 95% confidence intervals. Statistical significance was determined using pairwise comparisons of estimated marginal means (emmeans package; Tukey adjustment). Different letters indicate statistically significant differences among forager groups and phases of the foraging trip (*p* < 0.05). Sample sizes are shown in brackets.

## Data Availability

The original contributions presented in this study are included in the article/Supplementary Materials. Further inquiries can be directed to the corresponding authors.

## Supplementary Materials

The following supporting information can be downloaded at: https://www.mdpi.com/article/10.3390/insects17100980/s1, Figure S1: Sucrose responsiveness of resin, nectar, and pollen foragers. (A) Observed proportion of proboscis extension responses (PER) to increasing sucrose concentrations. (B) Estimated mean sucrose responsiveness scores obtained from the generalized linear mixed model. Different letters indicate significant differences among forager types (*p* < 0.05). Returning resin, pollen, and nectar foragers were captured at the hive entrance, individually harnessed, and tested for sucrose responsiveness following the protocol described for Experiment 1 of the main study. Sucrose responsiveness was analyzed using a generalized linear mixed model with a Beta Binomial family distribution implemented with the glmmTMB package because diagnostics indicated underdispersion with Binomial distribution. Forager type (resin, pollen, nectar) was included as fixed effect, whereas experimental day was included as random effect. Model assumptions were evaluated using the DHARMa package. Sucrose responsiveness differed among forager types depending on sucrose concentration, as indicated by a significant interaction between forager type and sucrose concentration (χ^2^ = 250.12, d.f. = 2, *p* < 0.0001). Pollen foragers were approximately 20 times more likely to respond than nectar foragers (OR = 20.1), and approximately 3.4 times more likely than resin foragers (OR = 3.40). Resin foragers in turn showed approximately six-fold higher response probability than nectar foragers (OR = 5.9; all *p* < 0.0001). Figure S2: Crop load of returning resin, pollen, and nectar foragers. Crop load (mg) measured in returning foragers after completing a foraging trip. Resin foragers consistently returned with very small crop loads compared with nectar and pollen foragers. Returning resin, pollen, and nectar foragers were captured at the hive entrance and processed according to the protocol described for Experiment 3 of the main study. Crops were dissected and weighed to determine crop load (mg), and, whenever crop contents were sufficient, crop sugar concentration (% *w*/*w*) was also measured. Returning resin foragers consistently exhibited extremely small crop loads (0.652 mg) compared with nectar (36.2 mg) and pollen foragers (4.79 mg). Resin foragers return to the colony carrying very limited crop contents despite collecting a non-nutritional resource transported externally in the corbiculae.
