# Supplementary material for: Crop Load Links Gustatory and Feeding Responses to Foraging Specialization in Honey Bees
Source: Insects. 2026 Sep 22;17(10):980. doi: 10.3390/insects17100980 (PMC13705172; doi:10.3390/insects17100980)
Supplement: Supplementary file 1 [file insects-17-00980-s001.zip › Figures S1 and S2.pdf]

Supplementary materials

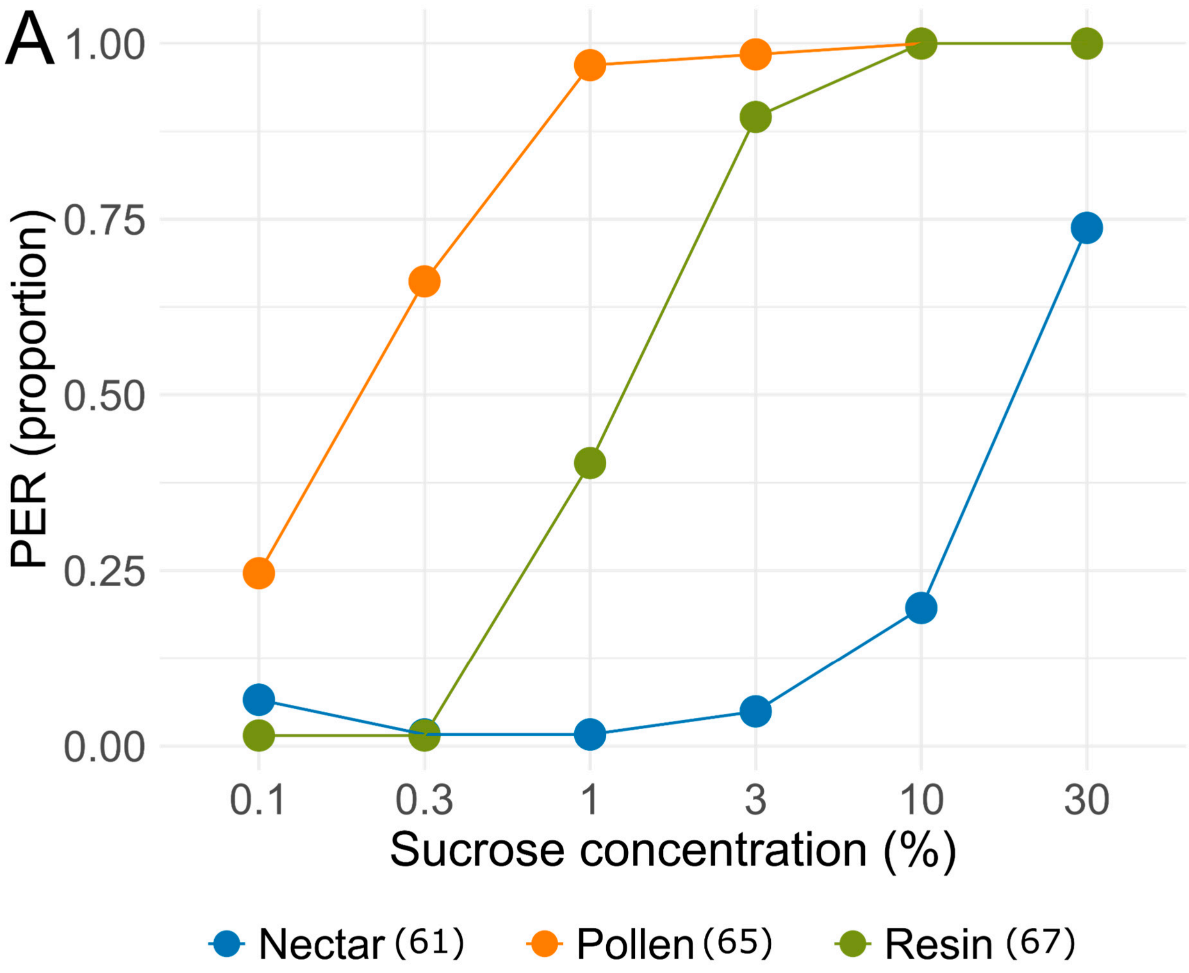

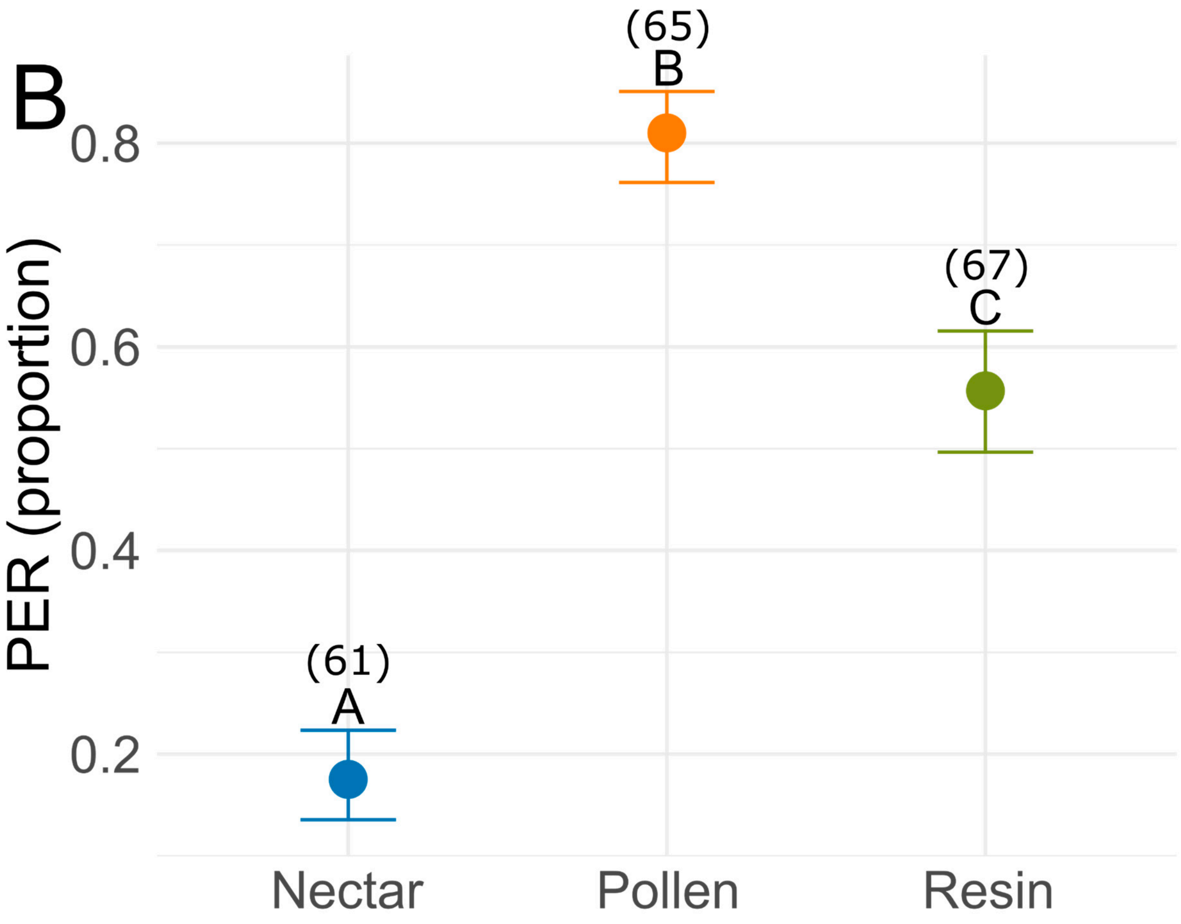

Figure S1. Sucrose responsiveness of resin, nectar, and pollen foragers. (A) Observed proportion of proboscis extension responses (PER) to increasing sucrose concentrations. (B) Estimated mean sucrose responsiveness scores obtained from the generalized linear mixed model. Different letters indicate significant differences among forager types ($P < 0.05$). Returning resin, pollen, and nectar foragers were captured at the hive entrance, individually harnessed, and tested for sucrose responsiveness following the protocol described for Experiment 1 of the main study. Sucrose responsiveness was analyzed using a generalized linear mixed model with a Beta Binomial family distribution implemented with the glmmTMB package because diagnostics indicated underdispersion with Binomial distribution. Forager type (resin, pollen, nectar) was included as fixed effect, whereas experimental day was included as random effect. Model assumptions were evaluated using the DHARMa package. Sucrose responsiveness differed among forager types depending on sucrose concentration, as indicated by a significant interaction between forager type and sucrose concentration ($\chi^2 = 250.12$, d.f. = 2, $P < 0.0001$). Pollen foragers were approximately 20 times more likely to respond than nectar foragers (OR = 20.1), and approximately 3.4 times more likely than resin foragers (OR =

3.40). Resin foragers in turn showed approximately six-fold higher response probability than nectar foragers (OR = 5.9; all $P < 0.0001$).

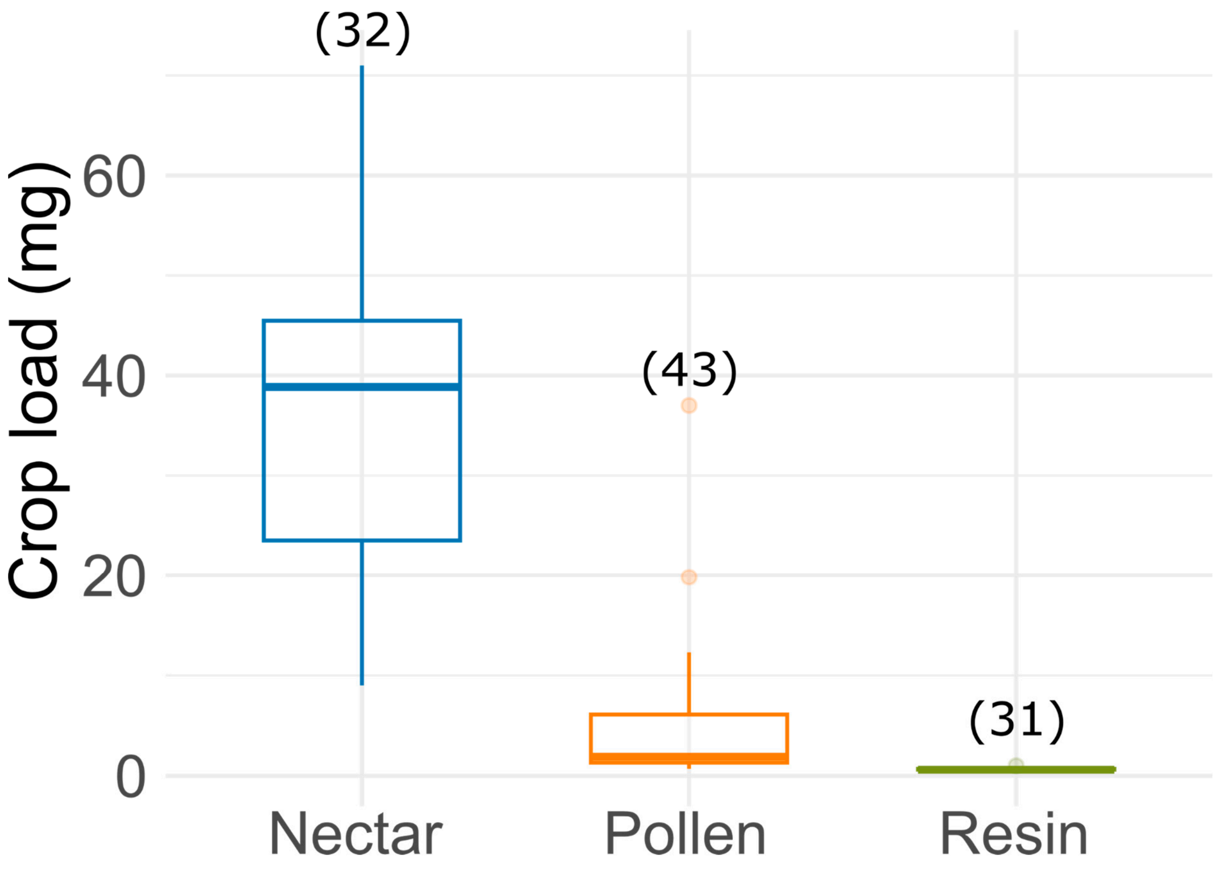

Figure S2. Crop load of returning resin, pollen, and nectar foragers. Crop load (mg) measured in returning foragers after completing a foraging trip. Resin foragers consistently returned with very small crop loads compared with nectar and pollen foragers. Returning resin, pollen, and nectar foragers were captured at the hive entrance and processed according to the protocol described for Experiment 3 of the main study. Crops were dissected and weighed to determine crop load (mg), and, whenever crop contents were sufficient, crop sugar concentration (% w/w) was also measured. Returning resin foragers consistently exhibited extremely small crop loads (0.652 mg) compared with nectar (36.2 mg) and pollen foragers (4.79 mg). Resin foragers return to the colony carrying very limited crop contents despite collecting a non-nutritional resource transported externally in the corbiculae.
